# Assessment of Water Quality in A Tropical Reservoir in Mexico: Seasonal, Spatial and Multivariable Analysis

**DOI:** 10.3390/ijerph18147456

**Published:** 2021-07-13

**Authors:** Juan G. Loaiza, Jesús Gabriel Rangel-Peraza, Antonio Jesús Sanhouse-García, Sergio Alberto Monjardín-Armenta, Zuriel Dathan Mora-Félix, Yaneth A. Bustos-Terrones

**Affiliations:** 1 División de Estudios de Posgrado e Investigación, TecNM-Instituto Tecnológico de Culiacán. Juan de Dios Batíz 310. Col. Guadalupe, 80220. Culiacán, SI, México; D18170809@culiacan.tecnm.mx (J.G.L.); jesus.rp@culiacan.tecnm.mx (J.G.R.-P.); zuriel.mf@culiacan.tecnm.mx (Z.D.M.-F.); 2 Departamento de Tecnología Ambiental, Universidad Tecnológica de Culiacán, Carr. Culiacán-Imala, Km 2, Los Ángeles, 80014. Culiacán, SI, México; a.sanhouse@utculiacan.edu.mx; 3 Facultad de Ciencias de la Tierra y el Espacio, Universidad Autónoma de Sinaloa. Circuito Interior Oriente, Cd Universitaria, 80040. Culiacán, SI. México; sa.monjardin12@info.uas.edu.mx; 4 División de Estudios de Posgrado e Investigación, CONACYT/TecNM-Instituto Tecnológico de Culiacán. Juan de Dios Batíz 310. Col. Guadalupe, 80220. Culiacán, SI, México;

**Keywords:** Adolfo Lopez Mateos Reservoir, comprehensive pollution index, spatial distribution graphs, multivariable analysis, PCA

## Abstract

Agricultural activities are highly related to the reduction of the availability of water resources due to the consumption of freshwater for crop irrigation, the use of fertilizers and pesticides. In this study, the water quality of the Adolfo López Mateos (ALM) reservoir was evaluated. This is one of the most important reservoirs in Mexico since the water stored is used mainly for crop irrigation in the most productive agricultural region. A comprehensive evaluation of water quality was carried out by analyzing the behavior of 23 parameters at four sampling points in the period of 2012-2019. The analysis of the spatial behavior of the water quality parameters was studied by spatial distribution graphs using the Inverse Distance Weighting interpolation. Pearson correlation was performed to better describe the behavior of all water quality parameters. This analysis revealed that many of these parameters were significantly correlated. The Principal Components Analysis (PCA) was carried out and showed the importance of water quality parameters. Ten principal components were obtained, which explained almost 90% of the total variation of the data. Additionally, the comprehensive pollution index showed a slight water quality variation in the ALM reservoir. This study also demonstrated that the main source of contamination in this reservoir occurs near sampling point one. Finally, the results obtained indicated that a contamination risk in the waterbody and further severe ecosystem degradations may occur if appropriate management is not taken.

## 1. Introduction

Water is the most important natural resource for life [1,2,3,4]. For this reason, this resource must be protected and any harmful influence on water bodies must be avoided [5,6,7,8,9]. The quality and quantity of available water resources are declining dramatically due to the increase of human activities and global warming; thus, the management of water resources has become more critical [10,11,12]. The water demand has increased due to the expansion of agriculture and it is well recognized that agricultural activities have caused the degradation of surface water quality around the world because of the use of a wide range of fertilizers and insecticides [12,13,14]. Extreme runoff events can lead to higher nutrient and particulate matter loads in reservoirs [15]. Nutrients enter the water body through the runoff of agricultural wastes that are rich in fertilizers containing nitrogen, phosphorus, and potassium [16]. The deterioration of water quality in reservoirs is a major concern issue since water availability depends on water quantity and quality. Consequently, water quality should be monitored [17,18,19]. Maintaining the water quality of water resources requires continuous monitoring of the physicochemical and bacteriological characteristics of the water [20,21,22]. By monitoring the water quality, it is possible to detect any variation that occurred, determining the reasons for such variations, and finding some possible solutions [23,24,25]. There could be many reasons why the water quality in a lake or reservoir is altered. Some waterbodies are controlled by climate and meteorological conditions. Lake Arrowhead is a good example; a dry season from the summer of 2012 to the winter of 2018 followed by intense storms in the winter of 2019 caused significant changes in the water quality of the lake [24]. In addition, land use has a great impact on water quality, such as urbanization. Changes in land use have a great impact on water bodies due to the slow resilient response of the aquatic ecosystems [26,27]. Lopes et al. [28] mention that other factors can influence water quality, such as thermal stratification, erosive rain events, the presence of natural biofilters, and landscape patterns in the surrounding areas. These water quality studies have identified key water quality variables that influence physical, chemical, and biological processes in water bodies [24,25,26,27,28].

In Mexico, several studies have been carried out on the health status of some water storage bodies. Muñoz-Nájera et al. [14] determined the spatial and temporal variability of the water quality parameters of the Tenango reservoir. They analyzed physicochemical parameters such as nutrients and metals. No significant changes were found from a spatial and temporal point of view, but the NO_3_^−^, NO_2_^−,^ and heavy metal concentrations indicated a poor water quality in the reservoir, which causes the water to be unsuitable for human consumption. Perez-Coyotl et al. [29] evaluated the concentrations of pollutants such as pharmaceutical drugs, personal care chemicals, organophosphate, organochlorine pesticides, and other persistent organic pollutants in the Madín reservoir located in Mexico City. This study found that the organic compounds (pesticides, pharmaceutical products, and metals) underwent biotic and abiotic transformations and acted as oxidizing agents that generated a redox imbalance in the aquatic ecosystem.

A previous study in the Adolfo Lopez Mateos (ALM) reservoir is reported by Quevedo-Castro et al. [30] who implemented a water quality index (WQI). Using multiparametric statistical tools, they analyzed 26 water quality parameters obtained from the year 2012-2017. The index described the water quality in the reservoir as ‘good’. The model identified that fecal coliforms, total phosphorus, organic nitrogen, and chlorophyll-a were the variables that showed the most influence on water quality in the ALM reservoir. This tropical water body is of great importance in the region because it is in an agricultural zone and supports intense fishing activity. However, in the last year, this reservoir has suffered from water scarcity due to the absence of rainfall and the intense agricultural activities in the surrounding area. These agricultural practices could be the main cause of the loss of regulatory ecosystem services, causing the loss of biodiversity and habitat in these aquatic ecosystems, this study focuses on a comprehensive evaluation of the water quality of the Adolfo Lopes Mateos reservoir. This study was conducted as a basic survey for the identification and location of water quality problems and their spatial distribution, by monitoring various water quality parameters at four sampling points within the ALM reservoir from 2012 to 2019, with the following objectives: to investigate the current state of water quality; to correlate the physicochemical and climatological parameters; to perform the PCA to identify seasonal changes in water quality and to identify possible sources of contamination in ALM reservoir based on the comprehensive pollution index. The results could be used to support water authorities’ decisions involved in managing water quality, controlling sources of pollution, and protecting water resources in the ALM reservoir.

## 2. Materials and Methods

### 2.1. Study Area

**Figure 1:**
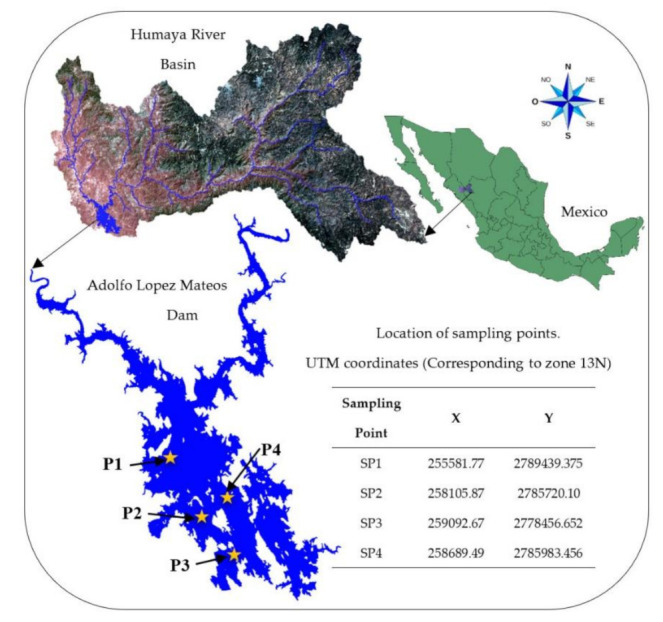
The geographic location of the study area.

The Humaya River basin is one of the most important basins in Mexico since it supplies water to the ALM reservoir. The Humaya River basin generates runoff at higher ground levels. This runoff is conducted through its main watercourse, the Humaya River, located in the municipality of Badiraguato, Sinaloa. The Humaya River flows from the north to the south (from SP1 to SP3). The ALM dam wall is located next to SP3. This reservoir is used to irrigate approximately 60,000 hectares of crops, which supports one of the most productive agricultural regions in Mexico: the Culiacan valley [18]. The ALM reservoir was built in 1957 for electric energy generation and irrigation purposes. This reservoir covers a surface area of 11,354 ha and has a storage capacity of 3,086.6 Mm^3^. The reservoir wall is 105 m in height. This reservoir can generate 90 MW of electrical energy. Fishing is practiced in this reservoir, where species such as tilapia, catfish, and bass can be found. Figure 1 presents the location of the ALM reservoir. The cartographic information was obtained from the National Institute of Statistics and Geography (INEGI).

### 2.2. Water Sampling and Analysis

Water quality in Mexico has been systematically monitored since 1973 by the National Water Quality Monitoring Network (RNMCA) [19], which is supervised by the National Water Commission (CONAGUA). CONAGUA is the Mexican water authority responsible for hiring qualified personnel and laboratories to monitor the water quality of the water bodies in Mexico. In this sense, the sampling, transportation, and preservation of samples meet the appropriate Mexican standards, and the samples are analyzed in an accredited laboratory by the Mexican Accreditation Entity, based on international standard methods for water analysis [31] and under strict Quality Assurance and Quality Control protocols.

The water quality data of the ALM reservoir were obtained semiannually from 2012 to 2019 and the samplings were carried out in 4 sampling points (SP) of the reservoir. The water quality parameters include Chlorophyll a (Cl-a), Fecal Coliforms (FC), Total Organic Carbon (TOC), Biochemical Oxygen Demand (BOD), Chemical Oxygen Demand (COD), Ammonia (NH_3_), Nitrates (NO_2_^-^), Nitrites (NO_3_^-^), Organic Nitrogen (N-ORG), Total Nitrogen (TN), Total Phosphorus (TP), Ortho Phosphates (O-PO_4_^3-^), True Color (TC), Transparency (Trans), Total Dissolved Solids (TDS), Total Suspended Solids (TSS), Turbidity (Turb), Redox Potential (RP), Electrical Conductivity (EC), Total Hardness (TH), pH, Dissolved Oxygen (DO), and Water Temperature (WT). The techniques used for the analysis of different parameters are provided in Table S1 in the supplementary materials. The climatological data were obtained daily in a monitoring station that is located at the reservoir wall.

### 2.3. Water Quality Assessment.

#### 2.3.1. Descriptive Analysis

A descriptive statistical analysis was performed to obtain Box and Whisker plots for each parameter analyzed. This descriptive analysis was carried out using the Statgraphics software.

#### 2.3.2. Spatial Analysis

The spatial behavior of the water quality parameters was represented using 60 data per year for each water quality parameter in 4 sampling points for a period from 2012 to 2019. The spatial analysis was supported by using the Inverse Distance Weighting (IDW) interpolation method, which was obtained with the Qgis 3.18 software. The weights used in the IDW method were calculated according to the weighting strategy proposed by [32]. These weights values are determined by the distance between sampling points according to Equation 1:

(1) $$z_{x,y}=\frac{\sum_{i=1}^{n}z_{i}d_{x,y,i}^{-\beta }}{\sum_{i=1}^{n}d_{x,y,i}^{-\beta }}$$

where *z_x,y_* is the water quality parameter to be estimated; *z_i_* represent the water quality value monitored at the *i^th^* sample point; *w_i_* is a weight that determines the importance of the water quality value monitored (*z_i_*) in the interpolation procedure; *d_x,y,i_* is the distance between *z_x,y_* and *z_i_*; and *β* is a coefficient defined by the user. In this study, the default value of 2 was used for coefficient *β*.

Likewise, an analysis of variance (ANOVA) (Statgraphics Technologies Inc, The Plains, USA) was carried out to figure out the spatial variation between the different parameters at a 95% confidence interval. The use of these statistical techniques better explains the behavior of water quality data and the most affected sites of the reservoir were identified.

#### 2.3.3. Temporal Analysis

Water quality parameters were depicted versus the sampling time. The water quality time series analysis was included to figure out possible temporal trends in the ALM reservoir. Then, a spectral analysis was performed to isolate regular water quality oscillations from random fluctuations. The spectral analysis depends on a visual inspection of graphical displays (periodograms) to detect the presence of certain periodicities in the water quality data. The spectral analysis was based on the finite Fourier transformation, which was used to decompose the data series into a sum of sine and cosine waves of different amplitudes and wavelengths [33]. Periodograms were depicted from the Fourier coefficients (*I(f_i_)*) against frequency (*f_i_*). The periodograms can be obtained by using Equation 2.

(2) $$I\left(f_{i}\right)=\frac{2}{N}\left\{{\left[\overset{N}{\underset{k=1}{\sum }}y\left(k\right)\cos\left(2\pi f_{i}k\right)\right]}^{2}+{\left[\overset{N}{\underset{k=1}{\sum }}y\left(k\right)\sin\left(2\pi f_{i}k\right)\right]}^{2}\right\}$$

for *i* = 1, 2, …], *q*; where *q* = (*N*-1)/2 for odd *N* and *q* = *N*/2 for even *N*; and *fi* = i/*N* is the *i^th^* harmonic of the fundamental frequency (1/*N*) up to the Nyquist frequency of 0.5 cycles per sampling. Since *I(f_i_)* is obtained by multiplying *y(k)* by sine and cosine functions, large values (sharp peaks) are obtained when this frequency coincides with a periodicity of this frequency occurring in *y(k)*.

#### 2.3.4. Multivariate Statistical Methods

A Pearson correlation analysis was carried out to describe the relationship (or correlation) between these quantitative variables [34]. Then, Principal Component Analysis (PCA) was used in this study to identify the relationship between the original indicator variables and transform them into independent principal components [35,36,37,38]. PCA analysis simplified the statistical analysis through new variables (main components). The visualization of the result was carried out using the graphs performed with Statgraphics and RStudio software.

### 2.4. Comprehensive Evaluation of Water Quality

The comprehensive pollution index was applied to qualitatively assess the surface water quality in the ALM reservoir. Furthermore, this index has been applied to assess water quality in many studies [39,40,41,42,43]. The comprehensive pollution index was determined by Equation (3):

(3) $$P=\frac{1}{n}\overset{n}{\underset{i=1}{\sum }}\left(\frac{C_{i}}{S_{i}}\right)$$

where *P* is the comprehensive pollution index, *C_i_* is the concentration of the measured water quality parameter (mg/L), *S_i_* represents the guideline value of water quality (this reference was taken from the National Water Commission in Mexico for agricultural use) and n is the number of selected pollutants (BOD, COD, NO_2_, NO_3_, TN, and TP). Finally, the value of *P* was used to classify the level of quality of the ALM reservoir [44]. The comprehensive pollution index classifies a waterbody as follows: for level I (≤0.20) the classification is “Cleanness”; level II (0.21-0.40) is “Sub-cleanness”; level III (0.41-1.00) is “Slight pollution”; level IV (1.01-2.0) is “Moderate pollution” and level V (≥2.01) is “Severe pollution” [40]. This index is a simplified contamination index, which is also known as the Raw contamination index [6].

## 3. Results and Discussion

### 3.1. Hydroclimatological Conditions

**Figure 2:**
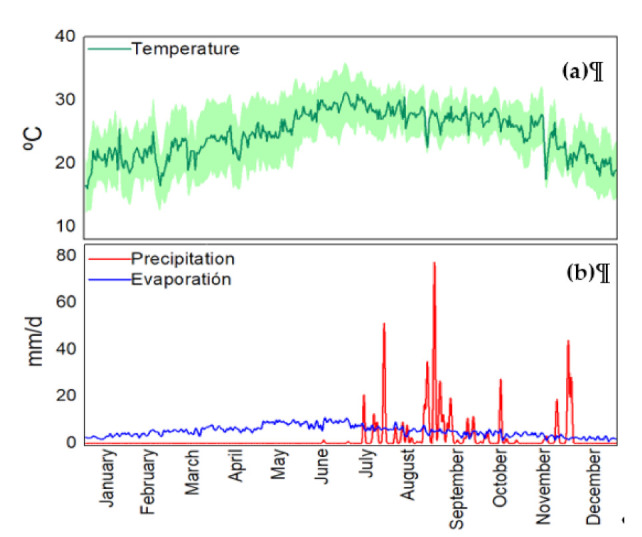
Hydrological and climatological data of the ALM reservoir. **(a)** Air temperature from 2012 to 2019. **(b)** Mean daily precipitation and evaporation from 2012 to 2019.

Hydrological and climatological features of the study area generate water quality variations in water bodies [45]. Therefore, these characteristics were also studied. This information was collected from the climatological and gauging stations located next to the ALM dam wall (close to SP3). Figure 2 presents the hydroclimatological behavior of the study area.

Figure 2a presents the behavior of mean air temperature in the study area from 2012 to 2019. The green line shows the mean daily temperature. The area covered in light green around the green line shows the minimum and maximum daily temperatures in the study area. This air temperature shows variations during the hydrological cycle. From May to October, the air temperature increases, and therefore higher evaporation is observed. Precipitation occurs from July to November, which is typical hydrological behavior in tropical zones. The average water temperature during a year was 29°C. From May to October, the ambient temperature is high, which causes an increase in the water temperature. In this period, the water temperature on the surface was between 27-31°C. This water temperature is higher than other tropical waterbodies reported in the literature [12,14]. Some studies suggest that temperature, evaporation, and precipitation are important parameters in a waterbody because these seasonal changes are directly related to water quality variations [22,46,47,48]. Seasonal water quality variations are related to runoff, evapotranspiration, drought, and floods processes [10], which modify the transformation and transport processes of nutrients and other substances in these aquatic environments [49]. Temperature is a critical parameter of water quality and a major determinant of the presence and productivity of aquatic organisms [50].

Figure 2b shows the behavior of mean daily precipitation and evaporation during a year. The mean daily precipitation and evaporation were calculated from 2012 to 2019. This figure shows that precipitation occurs during the rainy season, which is from July to November. The evaporation presents a slight increase from March to July, just during the dry season. During the dry period, the ALM reservoir shows its lowest water level.

### 3.2. Water Quality Assessment.

#### 3.2.1. Descriptive Analysis.

**Figure 3:**
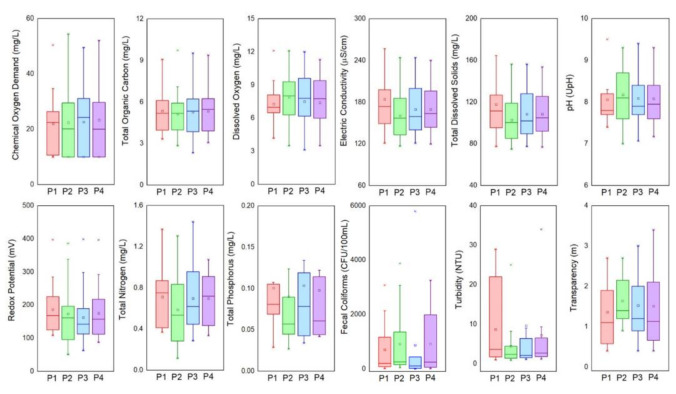
Box-and-whisker plots for water quality parameters at the 4 sampling points.

Tables S2 and S3 show the data obtained from annual samplings during the dry and rainy periods, respectively. A slight water quality variation is observed in most of the parameters studied during the 2012-2019 period. This situation can be related to low-intensity precipitation observed in the study area. The ALM reservoir does not receive wastewater discharges and most of the pollutants enter the ALM reservoir through runoff. Figure 3 presents the Box-and-Whisker plot for the water quality parameters between 2012 and 2019 at the four sampling points. This figure represents the distribution of the water quality data.

The organic matter load in the ALM reservoir was determined by the COD, BOD, and TOC parameters. When organic matter enters water bodies, the dissolved oxygen in the water decreases because it is used for organic matter decomposition [14]. The organic matter found, expressed as COD (22.6 mg/L), BOD (4.22 mg/L), and TOC (2.5 mg/L), was below the permissible limits of local standards (Table 1). The COD in the ALM reservoir is higher compared to other water bodies [22,51], while the BOD and TOC concentrations were very similar to what is reported in other studies [22,52]. Since the ALM reservoir is in a mountainous area and sparsely populated, organic matter in this water body is highly related to agricultural runoff. No domestic or industrial residual discharges have been identified in the basin.

On the other hand, turbidity describes the reduction in water clarity caused by suspended particles. In the ALM reservoir, a turbidity value of 7.46 NTU was found, which is very similar to that reported by Loucif et al. [17], but higher turbidity values have been reported in other water bodies [12]. According to Inatius and Rasmussen [50], a high turbidity value can reflect an increase in algal biomass and organic matter. Transparency in water is an important parameter that characterizes the primary production in lentic water bodies. This parameter is measured by using the Secchi disk. The mean transparency value in the ALM reservoir is 1.5 m. Other studies present lower transparency values [51,52]. This parameter, like the turbidity of the water, is affected mainly by suspended solids.

The ALM reservoir has a total dissolved solids (TDS) concentration of 19.46 mg/L, which is much lower than the concentration of other bodies of water [1,16,34]. The total suspended solids (TSS) in the ALM reservoir are above the permissible limits of local standards (Table 1). The mean TSS value was 31.53 mg/L, but the local regulations suggest a maximum TSS concentration of 20 mg/L. The TSS concentration previously mentioned is a mean value during the period of 2012-2019. It is important to mention that in 2013, very intense rains occurred due to the presence of a hurricane (Manuel), which greatly altered the concentration of the water quality parameters, especially the TSS. In this year, TSS achieved a TSS concentration of 355 mg/L. If the data for this year is excluded, the TSS mean value would be 13.65 mg/L. This concentration is very small compared to those reported in other studies [12].

The electrical conductivity (EC) in the ALM reservoir is low (171.04 µS/cm) compared to other water bodies [1,12,17]. The mean hardness concentration was 71.52 mg CaCO_3_/L. Loucif et al. [17] report very similar concentrations, however, very high concentrations have also been found in other water bodies [1,14,16]. The mean pH of the ALM reservoir was 8.08, which is within the optimum range to support aquatic life. This pH value could be considered normal in water bodies [5,12,17,22]. In this study, the mean RP value was positive (174.7 mV) thus, oxidation conditions are mainly present in the ALM reservoir, which is desirable.

The nutrient loadings produce chemical and biological changes in water bodies and have a direct and detrimental effect on water quality [30]. Total nitrogen was found in concentrations ranging from 0.115 to 1.441, with a mean value of 0.67 mg/L. This value is similar to that reported by Wang et al. [52] and is much lower than that found in other water bodies [5,12]. This value reflects the low degree of contamination of the water, which is below the permissible limit in local regulations.

Regarding biological indicators, high concentrations of fecal coliforms in water bodies indicate a high risk to human health [54]. In this study, this parameter was found below the maximum permissible limit of the local regulations (Table 1). However, some water samples exceeded the permissible levels of fecal coliforms established according to Mexican standards and the regular standards of the Food and Agriculture Organization for United Nations for the quality of irrigation water. These concentrations are derived mainly from human and animal feces that reach surface waters by wastewater and/or agricultural runoff [17]. Another biological indicator is chlorophyll “a”, which has been widely used to evaluate the eutrophication of a water body. In this study, a mean chlorophyll “a” concentration value of 8.33 mg/m^3^ was found, which is below the reported in other water bodies [9,52].

#### 3.2.2. Spatial Analysis.

**Figure 4:**
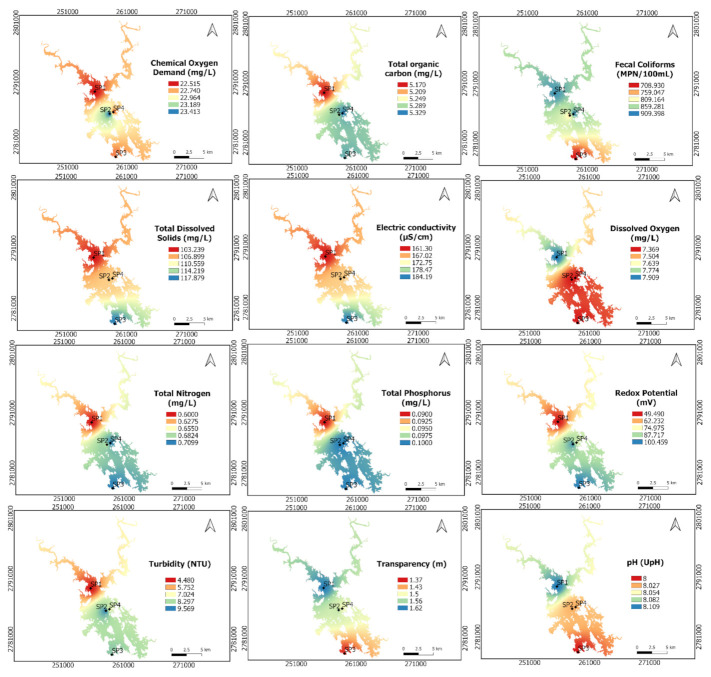
Spatial distribution of water quality parameters in the ALM reservoir using IDW interpolation.

Figure 4 presents the spatial behavior of the water parameters in the ALM reservoir. The concentration of COD was very similar in the four sampling points. Similar concentrations of TOC were found at SP1, SP2, and SP4, but a slightly lower concentration was found at SP3. Both EC and TDS showed higher concentrations in SP1 and lower concentrations in SP3. This is evidenced by the transparency of the water since greater transparency of the water was shown in SP3, which is located near the reservoir wall.

Regarding water nutrients, TP and TN showed higher concentrations at SP1 and SP2, while SP3 showed the lowest concentration in the ALM reservoir. The nitrogen/phosphorus ratio was used to define the limiting element in the growth of aquatic plants. When the N:P ratio is less than 9, nitrogen is the limiting element and when N:P>9 then phosphorus controls the growth of biomass. The N:P ratio was SP1=9.22; SP2=10.13; SP3=10.08 and SP4=10.38, which means that phosphorus controls the biomass growth. No spatial variation was found for the N:P ratio in the ALM reservoir.

The FC presented the lowest concentration in SP1 and the highest in SP3, which confirms the existence of wastewater discharges near the reservoir wall. On the other hand, DO presented a slight increase in SP3. However, DO values were similar in all sampling points. This situation is also observed with the pH because very similar values were observed throughout the ALM reservoir. The highest turbidity was present in SP1, possibly because this sampling point is closer to the water inflows to the ALM reservoir.

Based on the spatial distribution graphs (Figure 4), SP1 showed the highest concentration in the physicochemical parameters. SP1 is the furthest sampling site from the reservoir wall; therefore, it can be supposed that the water quality variation of the ALM reservoir highly depended on runoff.

#### 3.2.3. Temporal analysis.

**Figure 5:**
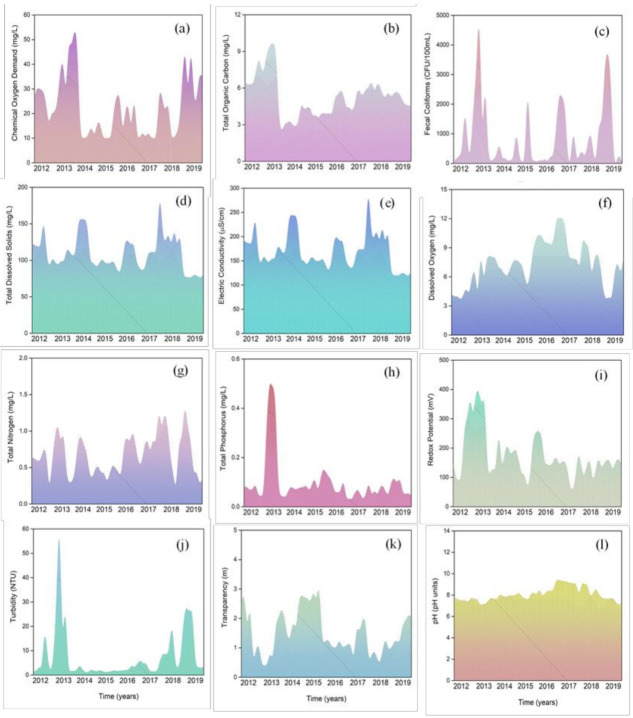
Seasonal behavior of water quality parameters in ALM reservoir.

Figure 5 presents the temporal behavior of the water quality parameters in the Adolfo Lopez Mateos (ALM) reservoir. According to the time series analysis, no trend was observed for the water quality parameters (neither increase nor decrease trends). Figure 5 suggests that the ALM reservoir responds to the hydrological and climatological variations of the study area. In particular, the occurrence of Hurricane Manuel significantly influenced some water quality parameters in 2013. In this year, a significant increase in the concentration of COD, TOC, TP was observed (Figure 5a, 5b, and 5h**,** respectively). The presence of organic pollutants in the reservoir was evidenced with COD and TOC. Figure 5a and 5b show that a maximum concentration of both parameters was reached due to the presence of a meteorological event. Figure 5a shows that the COD variation in the ALM dam is influenced by runoff, while Figure 5b shows the TOC variation is less sensitive to these hydrological variations.

A high variation of FC is noticed in the ALM reservoir (Figure 5c). Fecal coliform density peaks are observed throughout the study period, a situation that could be related to punctual wastewater discharges into the dam. Despite the mean value of FC was found below the limit permissible by the water authority, the coliform densities exceeding these limits are evident during the study period.

Figure 5d and 5e demonstrate that the behavior of TSS and EC are similar. Hence, most of the total dissolved solids are related to the presence of major ions. Figure 5f shows a slight variation of dissolved oxygen throughout the study period, with acceptable oxygen conditions in the ALM reservoir. The highest value of dissolved oxygen (12.1 mg/L) was observed in 2016. The supersaturation conditions of dissolved oxygen in the ALM reservoir suggest a eutrophication process due to the production of oxygen by algae [7]. The lowest concentration of dissolved oxygen (3.14 mg/L) was observed in 2018 and coincides with a period of low precipitation in the area.

The TN showed seasonal behavior and responds to runoff from the basin. According to Figure 5g, the maximum TN values coincide with the rainy season and the minimum TN values were observed precisely during the less rainy years in the study period. The behavior of the TP is also seasonal (Figure 5h). However, the TP seasonal variation is not as evident as the observed for TN due to the low TP concentration registered over time in the ALM reservoir. According to this, phosphorus is a key element in the growth of algae.

According to Figure 5l, pH showed a constant behavior from 2012 to 2019. This parameter was not influenced by any meteorological or climatological event, or by the entry of particulate material from the basin. This situation demonstrated a good buffer capacity of the ALM reservoir. These stability conditions were similar for RP, where oxidation conditions (positive RP values) were observed in the ALM reservoir over time. Figure 5i shows that this parameter was highly affected by the meteorological event that occurred in 2013. Due to the entry of a large amount of water into the reservoir, the oxidation conditions were enhanced. However, time series analysis showed a sudden change in oxidation conditions due to the oxidation of the organic matter from eroded soil.

Finally, Figure 5j and 5k show that Turbidity and Transparency are parameters that show a strong relationship. When the Turbidity showed higher values, the transparency showed the lower values and vice versa. Therefore, an inversely proportional relationship between these parameters is observed.

**Figure 6:**
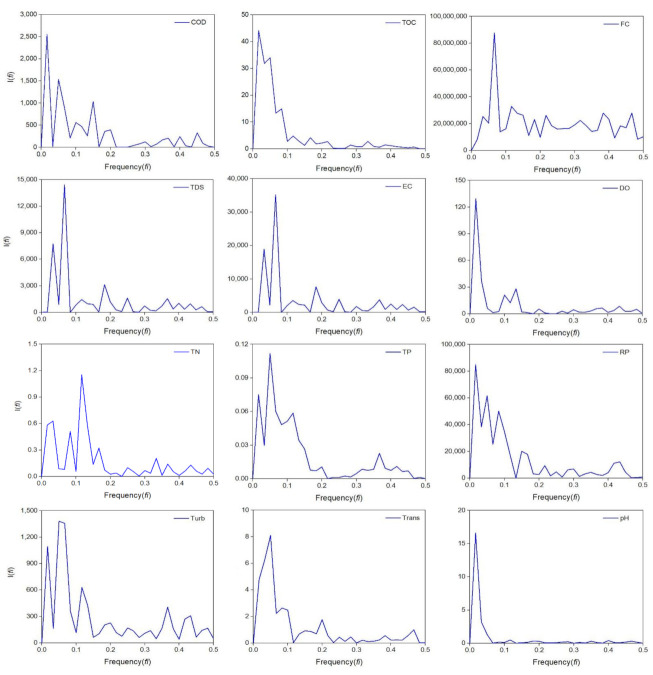
Periodograms of water quality parameters in ALM reservoir.

Figure 6 shows the periodograms of the water quality parameters analyzed in the time series. A periodogram is a graphical tool based on spectral analysis that describes periodicities in a time series. The spectral analysis calculates the values of the frequencies that correspond to the periods produced by the oscillatory behavior of the water quality parameters. A remarkable amplitude concerning the rest of the amplitudes could be considered as a significative frequency, which is evidenced with high peaks in the periodogram [55].

The periods of these oscillations can be obtained from periodograms through the inverse value of the frequency to the remarkable peak (*N*= 1/*f_i_*). In a time series, the oscillatory behavior is related to two components: cycle and seasonality. In a periodogram, a cycle is characterized by long periods (years) with a remarkable peak but relatively low amplitude. In contrast, seasonality produces remarkably high amplitudes but shorter periods. The spectral analysis applied to water quality time series is very important since significant cyclical variations in the water quality conditions of the ALM reservoir were identified (cycles). This analysis also quantified how regularly these variations occur (seasonality). Figure 6 also shows that most of the water quality parameters showed a periodicity in the range of 30 to 60 months. This situation demonstrates that the water quality parameters in the ALM reservoir respond to the hydrological and climatological variations present in the study area. This situation is desirable and suggests that the behavior of the water quality of the ALM reservoir has reached a stable condition. This condition allows subsequent water quality modeling using mathematical or stochastic models.

The spectral analysis carried out for the water quality time series in the ALM reservoir demonstrated that COD, TDS, EC, TN, TP, and Turb showed a cyclical and seasonal behavior. The periodograms for these water quality parameters identified the presence of two remarkable peaks. From these graphs, a seasonal period was estimated, which did not exceed 12 months for all the parameters. These results indicated that the behavior of these parameters responds to the variation of a complete hydrological cycle in the study area. The behavior of TN is of particular interest, where a seasonal periodicity of seven months was identified. This parameter is influenced by hydrological processes, such as dilution, runoff, and evaporation processes, but also depends on biological processes which accelerated the demand of this nutrient and reduced this seasonality.

The water quality parameters that only showed the cycle but did not show seasonality could be related to external pollution sources, such as the case of FC. Besides, the accuracy of the spectral analysis depends on the sampling periods of the original data. Some studies have reported diurnal seasonality in parameters such as water temperature, DO, and pH, which are associated with biological processes of oxygen production and consumption and CO_2_ absorption [56,57]. In this sense, the present spectral analysis was limited because the sampling period for the water quality data is every six months. Therefore, shorter water quality sampling periods are recommended to perform spectral analysis with greater accuracy.

#### 3.2.4. Multivariate Statistical Analysis

**Figure 7:**
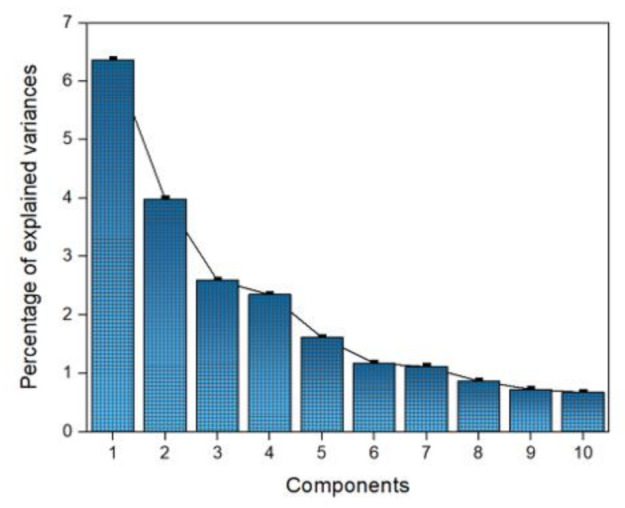
Percentages of the explained variances for each component.

The Pearson correlation diagram is included in Figure S1, as part of the Supplementary material. This figure shows the Pearson correlations between each pair of variables. The size of the circles indicates the relationship between the factors. Larger circles denote more similar correlations. Due to the large number of correlations found in the present study, the PCA was also performed in this study. With the implementation of the PCA and Pearson correlation analysis, a clearer panorama of water quality conditions is described in the ALM reservoir. The purpose of the PCA was to obtain a reduced number of linear combinations of the 24 water quality parameters (variables) that explain the greater variability in the data [38]. As a result of the PCA, 10 principal components explain almost 90% of the total variation of the data (see Figure 7). Table 2 shows the principal components (PC) for the water quality parameters of the ALM reservoir. The positive and negative values denote the influence of these components.

The first component explains 26% of the total variation of the data. This first principal component (PC) is highly related to the nutrients in the water such as NO_3_^-^ (0.35154), NO_2_^-^ (0.2161), TP (0.3451), and turbidity (0.3452). This component evidences the agricultural influence located in the surrounding area, which can be the cause of eutrophication in the reservoir. PC2 was represented by TDS (0.4179), EC (0.4179), and the water temperature (0.4117). This component is related to the influence of geochemical characteristics of major elements in soils. A cumulative variation percentage of 54% is represented with PC3, which is comprised by the TN (0.3824), N-Org (0.4833), and the BOD (0.3514), which could be associated with organic matter sources. PC4 explains 9.8% of the total variation and is related to pH (0.4144), DO (0.4413) and FC (0.2836). The PC5 presented a cumulative variation percentage of 70%. Based on the water quality parameters that comprise the principal components obtained from PCA, the influence of anthropogenic activities surrounding the study area is evidenced.

**Figure 8:**
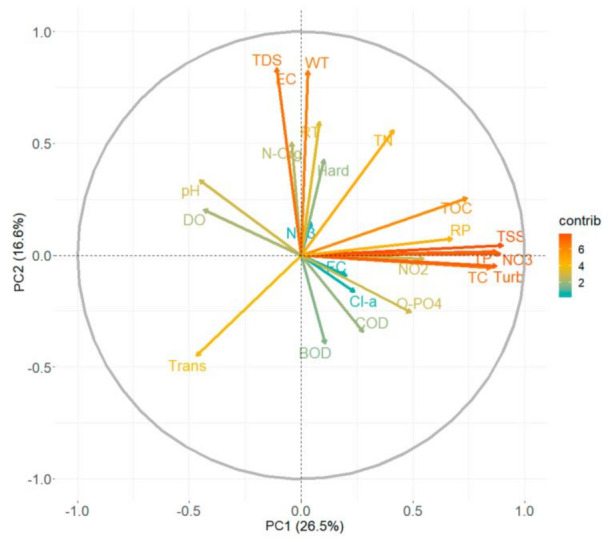
Principal Component Analysis diagram of water quality parameters.

Figure 8 shows a biplot of the components selected as the best representation of the variance of the data. The x-axis denotes PC1 and the y-axis denotes PC2. This figure shows the association of the 23 water quality parameters in the first two components, which represented 43% of the total variation (PC1 = 26.5% and PC2 = 16.5%). Parameters in the same direction are considered corresponding variables. In this figure, the contribution (importance) of the nutrients, such as NO_3_^-^, NO_2_^-^, and TP in the water quality is evident. This analysis also identifies groups of water quality variables that are linked to each other.

### 3.3. Comprehensive Evaluation of Water Quality

The comprehensive pollution index was applied to qualitatively evaluate the quality of surface water in the ALM reservoir. This index was evaluated for each sampling point classified by two seasons (dry season and rainy season). Table 3 presents the results obtained using this index. According to these results, almost all the sampling points can be classified as “Sub-cleanness”, except for SP2 during the rainy season, where a “Slight pollution” classification was obtained.

Based on the results obtained, the comprehensive pollution index (*p*) values showed a slight variation between sampling points and hydrological seasons studied. *p* values ranged from 0.30 to 0.42 were obtained. According to this, the ALM reservoir presents slight contamination. The highest *p* values were found in the rainy season and were related to pollutants transport into the reservoir by runoff. In this study, the COD was the most important parameter of the comprehensive pollution index because this parameter increased the *p* values. These results indicate the deterioration of the water quality in the ALM reservoir under the influences of pollution loading and the hydrological cycle. One possible source of water contamination is runoff from agricultural land in the rainy season. The intense fishing activity observed near SP2 could be another polluting source in the ALM reservoir since diesel boats are used for transportation.

Despite this situation, the comprehensive pollution index values obtained in ALM were low in comparison to other studies such as the report by Tibebe et al. [39], who obtained values from 0.68 to 0.98 for Lake Tana in Ethiopia. Using this index, Shi et al. [40] and Zhao et al. [41] demonstrated contamination of Lake Wuliangsuhai and Lake Baiyangdian, respectively. Shi et al. [40] discovered that the drainage of agricultural lands was the main source of pollutants in Wuliangsuhai Lake, China. They also report that the water quality is highly influenced by effluents from diffuse sources and by the many tourist attractions around the Lake. Zhao et al. [41] found that the water quality of the Baiyangdian Lake was influenced by sewage received from the Tanghe River reservoir. The pollutants were related to agricultural and domestic sewages, particularly the dispersed and unsettled wastewaters from local villages. Tourist attractions with diesel boats used as the primary means of transportation are mentioned as a source of pollutants for this water body. Therefore, strict controls on the external nutrient loading and hydrological regulations should be considered for water quality management.

## 4. Conclusions

The water quality of the Adolfo López Mateos reservoir was evaluated using data from 23 water quality parameters at four sampling points during eight years (2012-2019). Despite the spatial statistical analysis (ANOVA) showing that there was no significant variation in the water quality data from a spatial point of view, the dispersion graphs showed higher water quality values in SP1, which is located upstream. This spatial behavior of the water quality parameters demonstrated that the water quality in the ALM reservoir is highly influenced by anthropogenic induced and natural processes, such as agricultural runoff and erosion processes.

According to temporal analysis, the ALM reservoir responds to the hydrological and climatological variations of the study area. The occurrence of Hurricane Manuel significantly influenced some water quality parameters in 2013. The spectral analysis demonstrated that COD, TDS, EC, TN, TP, and Turb showed seasonal behavior, which did not exceed 12 months for all the parameters. Hence these parameters were mainly influenced by hydrological processes, such as dilution, runoff, and evaporation processes.

Principal component analysis showed the importance of the water quality parameters. The first component explains 26% of the total variation of the data and is highly related to the nutrients in the water such as NO_3_^-^, NO_2_^-^, TP, and turbidity. This component evidences the agricultural influence located in the surrounding area, which can be the cause of eutrophication in the reservoir.

The comprehensive pollution index was evaluated for each sampling point during the dry and rainy seasons. According to these results, the ALM were classified as “Sub-cleanness”, except for SP2 during the rainy season, where a “Slight pollution” classification was obtained. One possible source of water contamination is runoff from agricultural land in the rainy season. The intense fishing activity that takes place near SP2 could also be related to this classification. These findings can be used as support for the development of specific strategies to ensure reservoir water quality.

This work can be used as a reference for future works. For instance, due to the large number of correlations found in the present study, satellite images are intended to be used for the assessment of water quality in the ALM reservoir. Future works could also be aimed at the water quality modeling of this waterbody under different hydrological and climatological scenarios.

## Figures and Tables

**Table 1:** Permissible limits of water quality parameters from local standards and comparison with other studies.

Parameters	Units	Local Regulations	Present Study (Mean Value)	Other Studies	Ref.
Chlorophyll a	mg/m^3^	-	8.33	29.19	[52]
				9.8	[9]
Total Organic Carbon	mg/L	25	5.2745	2.5	[53]
Biochemical Oxygen Demand	mg/L	75	4.22	3.73	[22]
				1.32	[5]
				6.01	[51]
Chemical Oxygen Demand	mg/L	100	22.6023	34.98	[51]
				301.76	[12]
				4.44	[52]
Ammonia	mg/L	20	0.6739	0.18	[22]
				0.26	[5]
Nitrates	mg/L	-	0.01	0.55	[14]
				0.44	[12]
				0.10	[52]
Nitrites	mg/L	-	0.08	0.82	[17]
				0.189	[51]
				0.005	[14]
Organic nitrogen	mg/L	40	0.48	4.184	[51]
Total nitrogen	mg/L	20	0.6739	1.49	[5]
				89.5	[12]
Total Phosphorus	mg/L	12	0.0981333	0.09	[5]
				0.157	[51]
				2.49	[14]
Ortho phosphates	µg/L	-	0.03	5.81	[17]
				0.03	[52]
Fecal coliforms	MPN/100 mL	1000	853.017	11,000	[54]
True color	Pt/Co	-	19.41	-	-
Transparency	m	-	1.50817	0.55	[52]
Total dissolved solids	mg/L	-	109.467	1383	[1]
				246.69	[52]
				500	[16]
Total Suspended Solids	mg/L	20	31.53	17.2	[22]
				101.94	[12]
Turbidity	NTU	-	7.45967	9.58	[17]
				77.11	[12]
Redox potential	mV	-	174.705	90–350	[27]
				101.94	[12]
Electric Conductivity	µS/cm	-	171.045	1380	[17]
				1049.5	[12]
				2160.8	[1]
Total hardness	mg CaCO_3_/L	-	71.52	196.12	[14]
				920	[1]
				200	[16]
pH	UpH	6.5–8.5	8.08875	6.82	[22]
				7.72	[5]
				8.06	[12]
Dissolved oxygen	mg/L	-	7.47946	4.3	[22]
				0.69	[17]
				13.18	[14]
Water temperature	°C	35	27-31	21.5	[51]
				17.95	[12]

**Table 2:** Principal component (PC) loadings for the water quality parameters of the ALM reservoir.

Variable	PC 1	PC 2	PC 3	PC 4	PC 5	PC 6	PC 7	PC 8	PC 9	PC 10
Cl-a	0.093642	−0.08079	−0.256383	−0.11115	**0.442164**	**−0.37555**	0.165643	−0.198969	−0.233707	0.049873
FC	0.079252	−0.04591	0.161651	**0.283621**	0.217865	−0.09066	**0.293433**	**0.519364**	**−0.446964**	0.235624
TOC	0.293138	0.127541	−0.059318	0.038531	0.0538972	−0.28805	−0.091082	−0.1177	−0.067432	−0.11705
BOD	0.042001	−0.19675	**0.351427**	−0.223067	−0.185203	−0.14144	−0.109491	0.019477	**−0.334639**	0.003483
COD	0.109562	−0.17085	0.169393	−0.24709	−0.028079	0.111502	**−0.554146**	−0.025284	−0.183265	**0.428234**
NH_3_	0.017003	0.072504	−0.243513	0.0831134	**−0.470178**	**−0.33534**	−0.203399	−0.138189	**−0.448654**	−0.23007
NO_2_	0.216122	−0.00818	−0.25849	0.0593796	−0.13165	**0.456893**	0.15635	−0.098924	−0.216865	0.128716
NO_3_	**0.35154**	0.002797	−0.018794	0.0859606	−0.102378	0.224842	0.167402	0.003526	−0.015972	−0.06025
N-Org	−0.01671	0.251032	**0.483505**	−0.041062	0.099928	−0.13352	0.010985	−0.166192	0.190377	0.063674
TN	0.16313	0.279022	**0.382432**	0.0319605	−0.121184	−0.13178	0.026129	−0.217264	0.020167	−0.03942
TP	**0.345179**	0.007821	−0.109822	0.083719	−0.0823408	−0.00098	−0.12557	−0.104184	0.110346	0.235091
O-PO_4_	0.193558	−0.12779	0.0965129	−0.272364	0.0554454	−0.06736	**0.441099**	**−0.394754**	−0.043425	0.248274
TC	0.334995	−0.02744	0.136476	0.061004	−0.160985	0.005661	0.125513	0.266404	0.066588	−0.21787
Trans	−0.18421	−0.22460	−0.17536	−0.227029	−0.225287	−0.18441	0.267344	−0.026372	0.105945	**0.292235**
TDS	−0.04327	**0.41796**	0.003925	−0.24717	0.00611645	0.243933	0.097429	−0.036629	−0.193714	0.032304
TSS	**0.356304**	0.022048	−0.107402	0.100228	−0.0575834	0.018721	−0.117571	−0.056193	0.006652	0.243823
Turb	**0.345226**	−0.02429	0.192403	0.047943	−0.004730	−0.02786	0.048495	0.148968	0.0711072	−0.05476
RP	0.266409	0.036175	−0.160538	0.061433	**0.396313**	−0.07019	−0.181032	−0.142109	0.148797	−0.14274
EC	−0.04328	**0.41796**	0.003929	−0.247165	0.006119	0.243934	0.097421	−0.036628	−0.193708	0.03231
Hard	0.040492	0.212772	−0.083059	−0.361879	0.319424	−0.05047	−0.174383	0.278312	−0.203139	−0.12273
pH	−0.1785	0.166988	0.138901	**0.414444**	−0.0492071	−0.16648	0.124452	−0.190678	−0.194629	0.13992
DO	−0.1717	0.101235	0.00265046	**0.441366**	0.220383	0.165802	−0.214601	−0.193923	−0.109769	0.270465
RT	0.032146	0.297811	−0.154259	−0.04490	−0.138326	−0.30507	−0.045245	**0.365422**	0.287046	**0.471987**
WT	0.012042	**0.411747**	−0.225532	0.008933	−0.162245	−0.11790	0.0704453	0.0234009	0.068275	0.029991
Eigenvalue	6.36643	3.98247	2.58991	2.34522	1.61355	1.17261	1.11478	0.866124	0.726191	0.672713
Explained variance (%)	26.527	16.594	10.791	9.772	6.723	4.886	4.645	3.609	3.026	2.803
Cumulative % of variance	26.527	43.12	53.912	63.683	70.407	75.292	79.937	83.546	86.572	89.375

Data is highlighted in **bold** to identify the importance (eigenvalues) of water quality parameters on each Principal Component (PC) obtained.

**Table 3:** Comprehensive pollution index (*p*) values obtained in ALM reservoir.

Sampling Point	*p* Value	Classification Water Quality
Dry season		
SP1	0.3397	Sub-cleanness
SP2	0.3104	Sub-cleanness
SP3	0.3825	Sub-cleanness
SP4	0.3058	Sub-cleanness
Rainy season		
SP1	0.3650	Sub-cleanness
SP2	0.4173	Slight pollution
SP3	0.3619	Sub-cleanness
SP4	0.3825	Sub-cleanness

## Supplementary Materials

The following are available online at https://www.mdpi.com/article/10.3390/ijerph18147456/s1, Figure S1: Pearson correlation coefficients between the water quality parameters; Table S1: Methodologies used to assess water quality, Table S2: Data obtained from annual samplings in the dry period., Table S3: Data obtained from annual samplings in the rainy season.
