# Supplementary material for: Assessment of Water Quality in A Tropical Reservoir in Mexico: Seasonal, Spatial and Multivariable Analysis
Source: Int J Environ Res Public Health. 2021 Jul 13;18(14):7456. doi: 10.3390/ijerph18147456 (PMC8305193; doi:10.3390/ijerph18147456)
Supplement: Supplementary file 1 [file ijerph-18-07456-s001.zip › ijerph-1270353-supplementary.pdf]

Supplementary Material

Pearson Correlation Analysis

A Pearson correlation analysis was carried out to describe separately the behavior of each water quality parameter of the ALM reservoir and to study the relationship (or correlation) between these quantitative variables. For this analysis, all water quality data from the period of 2012-2019 at 4 sampling points were used. The analysis was performed using Origin 9.1 software and a Pearson correlation matrix was generated with RStudio.

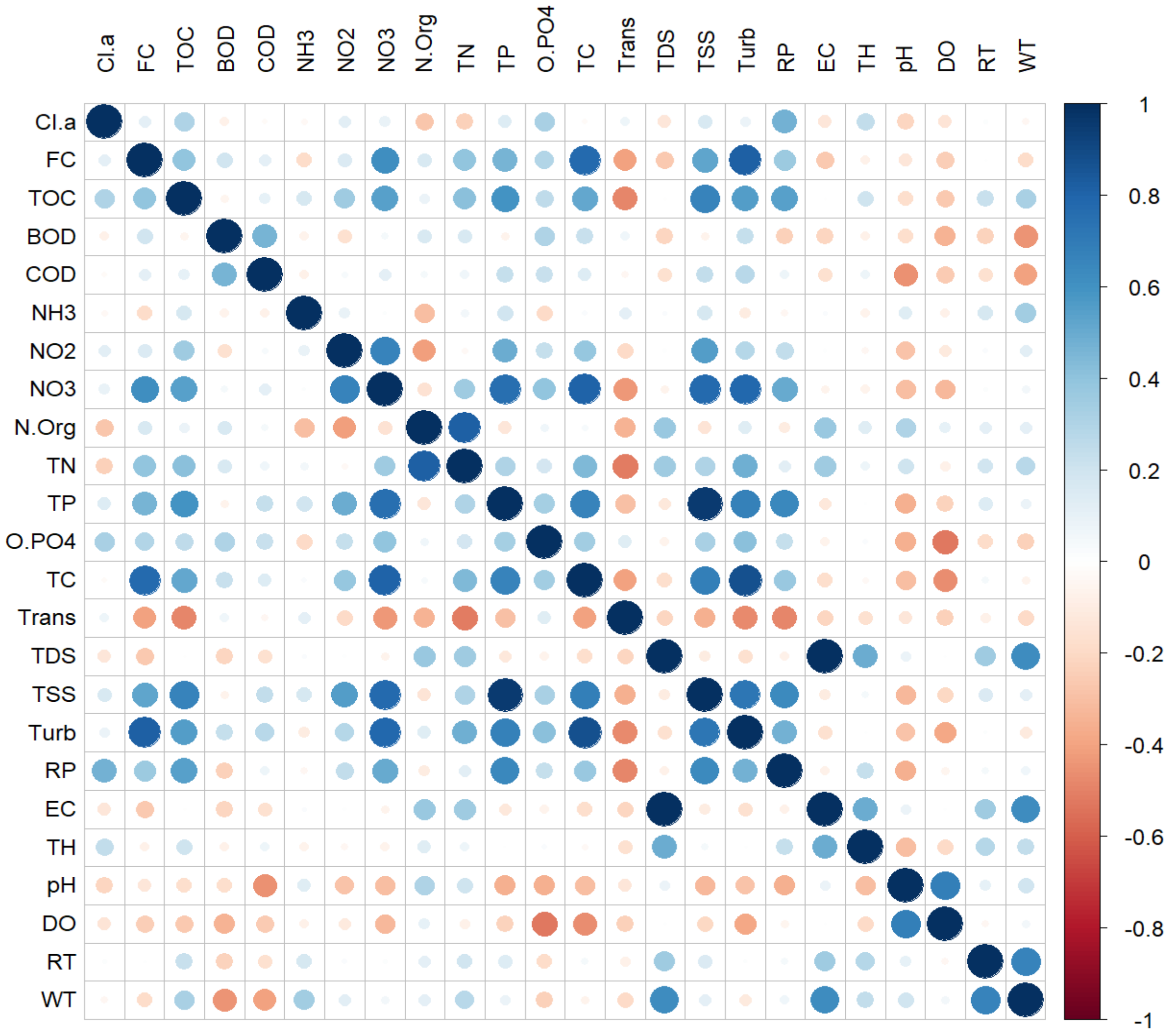

Figure S1. Pearson correlation coefficients between the water quality parameters.

Figure S1 presents the Pearson correlation diagram. This figure shows the Pearson correlations between each pair of variables. The range of this correlation coefficient is -1 to +1 and measures the strength of a linear relationship between two variables. The size of the circles indicates the relationship between the factors. Larger circles denote more similar correlations.

Pearson correlation analysis shows that a great amount of water quality parameters were correlated. Regarding the organic matter, it was observed that the total organic carbon correlates with NO_2^-, NO_3^-, TN, TP, O-PO_4^{3-}, TC, Trans, TSS, Turb, RP, DO, and WT. Biochemical oxygen demand is related to COD, O-PO_4^{3-}, DO, and WT, while the chemical oxygen demand showed a significant

correlation with Turb, pH, and WT. In turn, Total nitrogen was related to TP, TC, Trans, TDS, TSS, Turb, EC, and WT. Nitrates were correlated with NO_3^-, N-ORG, TP, TC, TSS, Turb, and pH, while nitrites showed a good Pearson correlation with TN, TP, O-PO_4^{3-}, TC, Trans, TSS, Turb, RP, pH and DO. Organic nitrogen is related to TN, Trans, TDS, EC, and pH. The total phosphorus was correlated with O-PO_4^{3-}, TC, Trans, TSS, Turb, RP, and pH, while the orthophosphates showed a relationship with TC, TSS, Turb, pH and DO.

Total dissolved solids correlated with TH and WT, and the total suspended solids with Turb, RP, and pH. Likewise, it was observed that fecal coliforms are correlated with TOC, NO_3^-, TN, TP, O-PO_4^{3-}, TC, Trans, TDS, TSS, Turb, RP, and EC. Also, Chlorophyll a is correlated with TOC, N-ORG, O-PO_4^{3-} and RP. The true color was related to the Trans, TSS, Turb, RP, pH, and DO, while Electrical conductivity is correlated only with TH and WT. The total hardness is correlated with the pH. Transparency is correlated

Data obtained from the samples of the ALM reservoir in the period 2012-2019.

The water quality parameters include: Chlorophyll a (Cl-a), Fecal Coliforms (FC), Total Organic Carbon (TOC), Biochemical Oxygen Demand (BOD), Chemical Oxygen Demand (COD), Ammonia (NH_3), Nitrates (NO_2^-), Nitrites (NO_3^-), Organic Nitrogen (N-ORG), Total Nitrogen (TN), Total Phosphorus (TP), Ortho Phosphates (O-PO_4^{3-}), True Color (TC), Transparency (Trans), Total Dissolved Solids (TDS), Total Suspended Solids (TSS), Turbidity (Turb), Redox Potential (RP), Electrical Conductivity (EC), Total Hardness (TH), pH, Dissolved Oxygen (DO), Room Temperature (RT) and Water Temperature (WT).

Table S1 presents the methodologies that were used to determine the quality parameters of the guide. **Table S2** presents the Data obtained from annual samplings in the dry period and **Table S3** presents the Data obtained from annual samplings in the rainy period.

Table S1. Methodologies used to assess water quality.

Parameters	Units	Method*
Chlorophyll a	mg/m^3	Spectrophotometric
Fecal coliforms	MPN/100 mL	9221
Total Organic Carbon	mg/L	5310
Biochemical Oxygen Demand	mg/L	5210
Chemical Oxygen Demand	mg/L	5220
Ammonia	mg/L	4500-NH_3
Nitrates	mg/L	4500-NO_3^-
Nitrites	mg/L	4500-NO_2^-
Organic nitrogen	mg/L	4500-N_{org}
Total nitrogen	mg/L	4500
Total Phosphorus	mg/L	4500-P
Ortho phosphates	µg/L	4500-P
True color	Pt/Co	**NMX-AA-045-SCFI-2001
Transparency	m	Secchi disk
Total dissolved solids	mg/L	2510
Total Suspended Solids	mg/L	2540
Turbidity	NTU	2130
Redox potential	mV	-
Electric conductivity	µS/cm	2510
Total hardness	mg $CaCO_3$/L	2340
pH	UpH	4500-H^+
Dissolved oxygen	mg/L	4500-O
Water temperature	°C	2550-B

* Standard methods for the examination of water and wastewater

** Mexican standards

Table S2. Data obtained from annual samplings in the dry period.

SP	Year	Cla	FC	TOC	BOD	COD	NH3	NO2	NO3	N.Org	TN	TP	O.PO4
1	2013	35.36	295.00	7.11	2.74	20.80	0.1069	0.0020	0.0065	0.0000	0.1154	0.0448	0.0251
1	2014	6.33	132.00	3.02	7.50	54.40	0.0820	0.0010	0.0110	0.2090	0.3030	0.0400	0.0140
1	2015	37.38	1274.00	4.35	4.69	16.08	0.0554	0.0121	0.0474	0.3742	0.4890	0.0895	0.0656
1	2016	11.59	122.00	3.98	2.00	28.29	0.0729	0.0007	0.0040	0.3631	0.4407	0.1238	0.0018
1	2017	7.66	2247.00	4.08	2.00	12.66	0.0326	0.0020	0.0301	0.4695	0.5342	0.0277	0.0073
1	2018	2.00	161.00	5.51	5.09	20.16	0.0195	0.0037	0.0332	1.2474	1.3038	0.0715	0.0390
1	2019	7.00	10.00	4.93	7.10	25.08	0.0446	0.0050	0.0204	0.3015	0.3715	0.0522	0.0241
2	2013	14.78	309.00	8.67	3.10	14.40	0.0457	0.0022	0.0095	0.7002	0.7576	0.0430	0.0053
2	2014	3.81	63.00	2.33	9.30	46.40	0.1220	0.0050	0.0400	0.1190	0.2860	0.0430	0.0210
2	2015	17.66	110.00	4.23	2.00	10.00	0.0836	0.0082	0.0332	0.2241	0.3491	0.0783	0.0733
2	2016	15.33	20.00	3.85	2.00	27.46	0.1646	0.0009	0.0046	0.3137	0.4838	0.1339	0.0020
2	2017	9.60	24196.00	4.53	2.00	10.00	0.0288	0.0018	0.0071	0.5087	0.5464	0.0342	0.0061
2	2018	3.00	439.00	6.70	2.00	25.20	0.0243	0.0023	0.0208	0.9119	0.9593	0.0397	0.0269
2	2019	2.00	336.00	4.55	2.00	36.86	0.0462	0.0075	0.0196	0.1816	0.2549	0.0547	0.0254
3	2013	29.63	2143.00	7.46	2.62	24.80	0.0679	0.0018	0.0057	0.6767	0.7521	0.0981	0.0080
3	2014	3.47	201.00	3.33	9.70	50.40	0.1190	0.0030	0.0310	0.2170	0.3700	0.0690	0.0210
3	2015	14.19	122.00	4.88	4.71	15.28	0.0897	0.0081	0.0413	0.2519	0.3910	0.0792	0.0991
3	2016	0.10	134.00	3.96	2.00	22.46	0.1989	0.0012	0.0040	0.3492	0.5533	0.1619	0.0046
3	2017	4.73	1266.00	4.26	4.90	10.13	0.0343	0.0014	0.0080	0.7379	0.7816	0.0288	0.0036
3	2018	0.82	279.00	6.01	6.07	32.11	0.0196	0.0021	0.0303	1.3168	1.3687	0.1076	0.0366
3	2019	6.00	10.00	4.54	2.00	34.88	0.0356	0.0050	0.0113	0.2855	0.3374	0.0477	0.0138
4	2013	12.54	1989.00	7.62	2.00	20.00	0.0590	0.0022	0.0108	0.4791	0.5511	0.0427	0.0053
4	2014	7.22	246.00	3.16	8.60	52.00	0.1420	0.0050	0.0340	0.2530	0.4340	0.0420	0.0140
4	2015	22.34	86.00	4.55	4.91	16.88	0.0562	0.0055	0.0507	0.4198	0.5322	0.0609	0.0518
4	2016	0.10	75.00	3.72	2.00	10.00	0.1200	0.0009	0.0040	0.2257	0.3507	0.1022	0.0010
4	2017	5.94	2046.00	4.41	2.00	10.00	0.0353	0.0013	0.0034	0.7582	0.7981	0.0552	0.0062
4	2018	0.95	657.00	6.51	2.00	26.04	0.0142	0.0035	0.0310	1.0254	1.0740	0.0420	0.0337
4	2019	3.00	10.00	4.65	2.00	24.24	0.0403	0.0050	0.0378	0.4006	0.4837	0.0522	0.0224

Table S2. Data obtained from annual samplings in the dry period (Continues).

SP	Year	TC	Trans	TDS	TSS	Turb	RP	EC	TH	PH	DO	RT	WT
1	2013	10.00	1.20	89.86	10.00	0.88	386.20	140.40	90.00	7.30		35.30	29.70
1	2014	7.50	2.40	104.96	10.00	1.50	134.80	164.00	129.80	7.60	8.00	33.00	25.80
1	2015	5.00	2.70	90.24	10.00	1.30	196.50	141.00	50.41	8.10	7.76	32.00	26.30
1	2016	15.00	1.30	81.28	10.00	1.90	258.10	127.00	62.59	8.70	10.40	34.00	27.00
1	2017	25.00	1.16	85.12	10.00	4.40	220.00	133.00	52.01	9.30	12.10	29.00	24.50
1	2018	10.00	0.95	124.16	10.00	8.20	95.50	194.00	66.37	9.00	9.30	36.00	30.80
1	2019	10.00	1.82	80.64	10.00	3.60	120.10	126.00	58.17	7.70	6.70	26.00	22.70
2	2013	15.00	0.90	101.95	8.00	5.00	298.10	159.30	154.00	7.60		35.50	32.40

2	2014	10.00	2.00	110.72	10.00	1.80	113.20	173.00	60.00	7.80	8.20	38.00	27.60
2	2015	5.00	2.80	98.56	10.00	1.80	189.60	154.00	50.41	7.90	7.57	31.00	27.80
2	2016	13.00	1.20	89.60	10.00	1.40	257.40	140.00	62.59	7.90	10.00	34.00	27.90
2	2017	18.00	1.12	89.60	10.00	6.40	142.60	140.00	52.01	9.40	12.00	31.00	26.20
2	2018	10.00	0.66	129.92	10.00	9.00	132.40	203.00	78.08	9.20	10.30	38.00	
2	2019	13.00	2.15	74.88	10.00	2.80	164.90	117.00	62.19	7.00	6.00	28.00	22.40
3	2013	20.00	0.40	164.48	55.50	22.00	279.70	257.00	184.00	7.50		35.30	31.80
3	2014	5.00	1.90	118.40	7.30	1.00	110.40	185.00	58.00	7.40	8.00	26.00	27.40
3	2015	5.00	2.70	93.44	10.00	2.30	174.30	146.00	54.15	8.00	6.68	29.00	27.50
3	2016	13.00	1.10	99.84	10.00	2.10	225.50	156.00	62.59	8.30	8.80	31.00	27.80
3	2017	18.00	1.05	95.36	10.00	4.60	176.40	149.00	52.01	9.50	12.10	32.00	26.90
3	2018	15.00	0.58	206.72	25.00	6.90	198.70	323.00	126.88	7.80	6.50	37.00	30.30
3	2019	15.00	2.03	80.00	10.00	3.30	144.70	125.00	54.16	7.40	8.00	27.00	23.30
4	2013	15.00	0.40	104.83	14.50	6.50	292.20	163.80	102.00	7.40		36.40	33.80
4	2014	5.00	2.00	108.80	10.00	2.00	112.30	170.00	68.90	7.60	8.00	35.00	26.20
4	2015	5.00	2.70	94.08	10.00	2.20	158.40	147.00	52.28	8.20	7.60	32.00	28.10
4	2016	13.00	1.10	92.16	10.00	1.80	249.90	144.00	64.68	7.80	10.20	33.00	27.70
4	2017	15.00	0.44	89.60	10.00	5.30	157.70	140.00	52.01	9.30	11.30	32.00	26.00
4	2018	15.00	0.66	139.52	12.00	9.30	145.70	218.00	78.08	9.00	9.40	36.00	33.30
4	2019	13.00	1.90	78.72	10.00	3.30	153.90	123.00	62.19	7.30	7.70	27.00	23.10

Table S3. Data obtained from annual samplings in the rainy season.

SP	Year	Cla	FC	TOC	BOD	COD	NH3	NO2	NO3	N.Org	TN	TP	O.PO4
1	2012.00	14.00	246.00	6.36	7.16	29.53	0.2277	0.0102	0.0225	0.3281	0.5885	0.0672	0.0171
1	2013.00	12.85	156.00	9.73	2.56	43.85	0.3668	0.0577	0.2472	0.1153	0.7870	0.4820	0.0474
1	2014.00	8.00	201.00	2.85	2.00	10.00	0.1490	0.0020	0.0160	0.6330	0.8000	0.0680	0.0390
e1	2015.00	0.10	3076.00	3.46	2.00	10.00	0.0969	0.0053	0.0043	0.1770	0.2835	0.0556	0.0060
1	2016.00	2.37	262.00	5.93	2.00	11.02	0.1599	0.0037	0.0025	0.6693	0.8354	0.0542	0.0047
1	2017.00	4.00	1355.00	5.32	4.21	10.00	0.2605	0.0029	0.1017	0.5643	0.9295	0.0269	0.0046
1	2018.00	5.00	3873.00	5.67	18.20	23.30	0.0487	0.0027	0.1530	0.8245	1.0289	0.0932	0.0516
2	2012.00	8.00	161.00	6.22	6.44	31.09	0.1078	0.0020	0.0163	0.4930	0.6191	0.0807	0.0721
2	2013.00	12.70	5794.00	9.53	3.76	36.54	0.1283	0.0315	0.5944	0.3949	1.1491	0.5232	0.0675
2	2014.00	1.90	109.00	2.91	2.00	10.00	0.1190	0.0050	0.0600	0.7720	0.9560	0.0810	0.0320
2	2015.00	2.72	1.00	3.86	2.00	10.00	0.1236	0.0055	0.0040	0.3148	0.4479	0.1223	0.0086
2	2016.00	2.84	41.00	5.38	2.00	24.59	0.0722	0.0037	0.0027	0.8352	0.9138	0.0606	0.0034
2	2017.00	2.00	10.00	5.59	5.29	10.00	0.4381	0.0014	0.0092	0.2185	0.6672	0.0530	0.0046
2	2018.00	4.00	3448.00	5.65	14.40	49.48	0.0469	0.0028	0.1674	1.2239	1.4410	0.1191	0.0547
3	2012.00	11.00	75.00	6.56	6.02	26.42	0.1324	0.0020	0.0058	0.5061	0.6463	0.0809	0.0809
3	2013.00	18.30	3076.00	9.07	2.00	23.55	0.1136	0.0273	0.5359	0.2356	0.9124	0.3492	0.0568
3	2014.00	6.64	759.00	3.35	2.00	10.66	0.0670	0.0040	0.1330	0.6610	0.8650	0.0770	0.0290
3	2015.00	7.96	75.00	3.60	2.00	10.00	0.0161	0.0037	0.0040	0.3883	0.4120	0.0914	0.0069
3	2016.00	5.00	160.00	5.35	2.00	22.05	0.1156	0.0023	0.0040	0.7476	0.8695	0.0567	0.0040

3	2017.00	6.00	10.00	6.03	2.00	12.60	0.5834	0.0015	0.0054	0.3222	0.9125	0.0728	0.0033
3	2018.00	6.00	1162.00	5.19	20.30	34.64	0.0477	0.0032	0.1770	0.6040	0.8318	0.1054	0.0556
4	2012.00	9.00	345.00	6.24	4.46	29.53	0.1811	0.0014	0.0040	0.4040	0.5905	0.0690	0.0152
4	2013.00	8.77	3255.00	9.38	2.50	24.36	0.1390	0.0095	0.5687	0.3430	1.0602	0.4819	0.0419
4	2014.00	2.84	63.00	3.07	2.00	11.48	0.1340	0.0030	0.0460	0.5360	0.7190	0.0740	0.0360
4	2015.00	8.00	41.00	3.91	2.00	10.00	0.0993	0.0017	0.0024	0.3084	0.4118	0.1143	0.0066
4	2016.00	3.00	203.00	5.46	2.00	29.68	0.0819	0.0024	0.0051	0.9443	1.0336	0.1205	0.0039
4	2017.00	8.00	10.00	5.99	5.31	10.00	0.3534	0.0017	0.0049	0.4077	0.7677	0.0472	0.0041
4	2018.00	8.00	3076.00	5.53	4.50	51.62	0.0439	0.0046	0.1667	0.6839	0.8992	0.1221	0.0774

Table S3. Data obtained from annual samplings in the rainy season (Continues).

SP	Year	TC	Trans	TDS	TSS	Turb	RP	EC	TH	PH	DO	RT	WT
1	2012.00	25.00	1.40	118.85	10.00	3.70	89.40	185.70	97.63	7.48	2.50	34.00	30.00
1	2013.00	30.00	0.90	100.48	295.00	6.10	338.40	157.00	79.84	7.40	9.31	35.60	32.60
1	2014.00	13.00	2.00	156.16	10.00	1.00	178.60	244.00	179.60	7.80	6.30	35.00	32.10
1	2015.00	25.00	2.50	95.36	6.00	1.40	61.40	149.00	52.99	7.50	4.80	38.00	31.00
1	2016.00	13.00	1.30	120.32	10.00	4.30	138.90	188.00	54.95	8.40	9.20	33.00	33.80
1	2017.00	13.00	1.96	112.00	10.00	1.50	51.20	175.00	53.42	9.10	7.80	35.00	33.40
1	2018.00	50.00	1.26	76.16	17.50	25.00	161.20	119.00	48.72	7.60	4.00	32.00	23.70
2	2012.00	20.00	3.00	120.13	10.00	1.50	92.40	187.70	85.43	7.65	3.93	33.32	28.00
2	2013.00	75.00	0.40	93.44	355.00	74.00	398.40	146.00	65.13	7.07	3.14	34.70	29.80
2	2014.00	25.00	1.30	156.16	13.00	2.10	115.40	244.00	128.60	7.90	6.90	35.00	32.00
2	2015.00	15.00	3.00	96.64	18.00	1.10	115.10	151.00	57.60	7.70	6.18	38.00	30.10
2	2016.00	10.00	1.00	128.00	14.00	2.00	145.90	200.00	44.77	8.40	9.60	34.00	33.80
2	2017.00	15.00	1.50	110.72	10.00	1.40	63.20	173.00	51.37	9.10	7.90	36.00	32.90
2	2018.00	40.00	1.20	77.44	33.08	29.00	148.10	121.00	50.66	7.70	3.60	31.00	23.80
3	2012.00	20.00	2.51	122.50	10.00	1.50	148.00	191.40	85.43	7.70	4.18	33.40	32.00
3	2013.00	50.00	0.40	96.00	185.00	29.00	397.20	150.00	84.04	7.70	7.04	34.30	30.00
3	2014.00	20.00	1.30	156.80	18.00	4.20	285.20	245.00	53.10	8.10	6.90	30.00	30.10
3	2015.00	25.00	2.50	103.04	17.00	1.40	108.80	161.00	71.42	7.80	7.32	36.00	29.70
3	2016.00	13.00	1.00	126.72	20.67	2.10	143.80	198.00	46.81	8.20	9.40	35.00	33.80
3	2017.00	18.00	1.80	111.36	10.00	1.70	137.50	174.00	53.42	9.20	8.10	33.00	31.80
3	2018.00	40.00	0.97	77.44	36.43	23.00	125.40	121.00	60.41	7.70	4.40	29.00	23.60
4	2012.00	25.00	2.74	117.44	10.00	1.80	97.50	183.50	95.19	7.50	3.59	34.00	33.00
4	2013.00	50.00	0.50	96.00	250.00	34.00	396.50	150.00	67.23	7.17	4.97	35.30	30.20
4	2014.00	15.00	1.50	153.60	7.00	1.20	218.00	240.00	55.10	7.90	6.00	32.00	31.70
4	2015.00	15.00	3.40	96.00	16.00	1.40	88.30	150.00	43.78	8.10	6.28	39.00	30.60
4	2016.00	13.00	1.00	125.44	12.00	2.70	146.40	196.00	44.77	8.40	9.50	34.00	33.80
4	2017.00	13.00	2.10	108.16	10.00	1.40	96.50	169.00	51.37	9.10	7.90	35.00	32.20
4	2018.00	40.00	1.13	76.80	38.00	29.00	157.30	120.00	52.61	7.70	3.50	32.00	23.70
